# The Prevalence of Overweight and Obesity in an Adult Kuwaiti Population in 2014

**DOI:** 10.3389/fendo.2019.00449

**Published:** 2019-07-09

**Authors:** Elisabete Weiderpass, Edoardo Botteri, Joseph C. Longenecker, Abdullah Alkandari, Rihab Al-Wotayan, Qais Al Duwairi, Jaakko Tuomilehto

**Affiliations:** ^1^International Agency for Research on Cancer, The World Health Organization, Lyon, France; ^2^Cancer Registry of Norway, Institute of Population-Based Cancer Research, Oslo, Norway; ^3^Norwegian National Advisory Unit for Women's Health, Women's Clinic, Oslo University Hospital, Oslo, Norway; ^4^Primary Health Care Department, Ministry of Health, Kuwait City, Kuwait; ^5^Faculty of Public Health, Kuwait University, Kuwait City, Kuwait; ^6^Research Sector, Dasman Diabetes Institute, Kuwait City, Kuwait; ^7^Department of Public Health, University of Helsinki, Helsinki, Finland

**Keywords:** overweight, obesity, body mass index, prevalence, Kuwait, Middle East, WHO STEPS survey

## Abstract

**Background:** According to World Health Organization (WHO) estimates, Kuwait is ranked amongst the top countries in the world in obesity prevalence. This study aims to describe the prevalence of overweight, obesity, and various types of adiposity in Kuwaiti adults.

**Methods:** This cross-sectional study of 3,915 Kuwaiti adults aged 18–69 years used the STEP-wise approach to surveillance of non-communicable diseases, a WHO Instrument for Chronic Disease Risk Factor Surveillance. We assessed demographic information, lifestyle, personal and family history of diseases and physical measurements (height, weight, waist, and hip circumferences). All participants with valid height and weight measurements (*n* = 3,589) were included in the present analysis. Overweight was defined as BMI 25–29.9 kg/m^2^ and obesity as BMI ≥30 kg/m^2^.

**Results:** Obesity prevalence was 40.3% [95% confidence interval, 38.6–42.0%] (men, 36.5%; women, 44.0%); and overweight prevalence was 37% [35.4–38.7%] (men, 42%; women, 32.1%). The median BMI was 28.4 kg/m^2^ among men and 29.1 kg/m^2^ among women. Obesity prevalence was directly associated with female sex, age, history of diabetes, and being married in both men and women; and was inversely associated with education level in women. The prevalence of elevated waist-to-hip ratio was 46.9% among men and 37.9% among women. Waist circumference, waist-hip and waist-height ratios were directly associated with diabetes in both men and women, and inversely associated with education level in women.

**Conclusion:** Almost eight in ten Kuwaiti adults were overweight or obese. Urgent public health action is warranted to tackle the obesity epidemic in Kuwait.

## Introduction

The epidemic of obesity does not show signs of halting in most countries of the world (1). Obesity and overweight are responsible for an estimated 4 million deaths globally and estimated to be increasing (2). The Eastern Mediterranean Region (EMR) in particular is heavily affected by the obesity epidemic and its consequences. The Global Burden of Diseases (GBD) 2015 collaborators estimate that prevalence of obesity in adults in the EMR increased from 15% in 1980 to 21% in 2015, which is far higher than the global average of 12% in the 2015. The prevalence of obesity in adults in Kuwait according to the GBD estimate is 41% in men and 49% in women in 2015; being second only to Qatar in leading the highest obesity prevalence rates within the EMR (3). Previously, the prevalence of obesity in Kuwaiti adults was reported to be 23% among men and 39% among women in the 1980s (3) and 36% amongst men and 48% amongst women aged 20–64 years in 2006 (4).

A nationally representative survey of adults was conducted in Kuwait in 2014 to assess the prevalence of non-communicable diseases and their risk factors (5). This study reports on the prevalence of overweight and obesity in Kuwaiti adults and its associated risk factors. In addition to body mass-index (BMI), we also describe for the first time data on other measures of adiposity in the Kuwaiti population.

## Methods

### Study Design and Sampling

This study analyzed data from the 2014 STEPS survey on non-communicable diseases (NCD) risk factors in Kuwait, which was supported by the Kuwait Ministry of Health (MOH) and the World Health Organization (WHO) (5). The STEPS survey methods have been previously described in detail (6). In brief, the target population of this cross-sectional survey included adult Kuwaiti nationals from all Kuwait governorates. The Kuwait Ministry of Information randomly sampled Kuwaiti citizens aged 18–69 years from the national civil registration rolls, with separate random sampling according to eight sub-groups stratified by sex and four age groups (18–29, 30–44, 45–59, and 60–69 years). The study estimated that 3,842 participants were needed to achieve a 5% acceptable margin of error within each stratum, assuming *Z* = 1.96, a 50% prevalence of a specific characteristic and equal representations in the eight strata. To account for incomplete data and non-responders, this sample selection estimate was increased by 12.5%, yielding a sample selection size of 4,391. The response rate for the questionnaire and physical examination steps of the study was 89% (*n* = 3,915; women, 96%; men, 80%). The analysis dataset included the 3,589 participants who had valid measurements of both height and weight. Of these, 307 were missing waist and hip circumference, so analyses of waist-hip ratio (WHiR) and waist-height ratio (WHtR) included 3,282 participants.

### Data Collection

The WHO STEP-Wise Approach to Surveillance survey methods were used, including a structured questionnaire (Step 1), physical examination (Step 2), and a blood draw (Step 3). Data from Steps 1 and 2 were used for the present analysis. The questionnaire, administered by face-to-face interview, included information regarding sociodemographic factors, tobacco smoking, exercise, and medical history, among other characteristics. The physical examination included measurement of weight (0.1 kg precision) and height (0.5 cm precision) using the Growth Management Scale, a device suitable for survey purposes (employing a weight scale and a height gauge with laser). BMI was calculated as weight (kilograms) divided by the height (meters) squared and classified according to the WHO cut-points for underweight (<18.5 kg/m^2^), normal weight (18.5–24.9 kg/m^2^), overweight (25.0–29.9 kg/m^2^), Class I obesity (30.0–34.9 kg/m^2^), Class II Obesity (35.0–39.9 kg/m^2^), and Class III obesity (≥40 kg/m^2^) (7). Weight and height for all those with an outlying BMI were checked for biological plausibility. Of 33 BMI values >50 kg/m^2^, 13 had biologically impossible combinations of weight and height; their BMI, weight and height were therefore recoded as missing. All those with BMI <17 kg/m^2^ had biologically plausible weight and height combinations. Waist circumference was measured using a MioTape device (a non-extensible tape measure, with ±1 mm precision), with the tape placed on the bare midriff midway between the lowest rib and the superior iliac crest while ensuring the tape was horizontal across the back. Bare hip circumference was measured using the same device placed at the maximum circumference of the buttocks. WHiR and WHtR were computed for all respondents consenting to the measurement except for pregnant women. Fourteen individuals had a WHiR ≤0.55 and 10 individuals had a WHiR ≥1.8, all of whom had biologically inconsistent waist and hip circumferences in relation to their weight and height. Therefore, waist and hip circumference and WHiR were coded as missing for these outliers. Cut-off values for elevated WHiR were ≥0.90 for males and ≥0.85 for females, according to WHO recommendations.

Teams composed of physicians, nurses, dieticians, social workers, and phlebotomists collected data at MOH primary healthcare centers within each governorate. The selected survey candidates were contacted and informed regarding the scope and purpose of the study, after which they were invited to come to the center for full informed consent and data collection procedures. Participants were enrolled from March 2014 to September 2014.

### Data Management and Statistical Analyses

Statistical analyses were conducted using SAS Statistical Package (version 9.4), including the package survey commands to account for the sampling weights and provide population-based estimate, and the STATA Statistical Package (version 14). Sample weights for each of the eight study strata defined by age (18–29 years; 30–44 years; 45–59 years, and 60–69 years) and sex were calculated as the product of the sample selection weight (population number/sample number), the non-response weight (1/response rate) and the population weight (population proportion/sample proportion). The Chi-square test, Chi-square test for trend and Mann-Whitney-U test were used to assess the statistical significance of sex (men vs. women) with categorical, ordinal and continuous variables, respectively (Table 1). Statistical significance for the trend of continuous anthropometric measures across the four age groups (Table 2) was evaluated using univariable linear regression models, with the anthropometric measure as the dependent continuous variable and age group entered as a continuous independent variable.

**Table 1:** Characteristics of the study population, overall and by sex (unweighted analyses).

**Characteristic**	**Total (*****n*** **= 3,589) 100%**		**Men (*****n*** **= 1,381) 38.5%**		**Women (*****n*** **= 2,208) 61.5%**		***p*-value^**a**^**
	***n***	**(%)**	***n***	**(%)**	***n***	**(%)**	
Age (years)							
≤25	686	(19.1)	304	(22.0)	382	(17.3)	0.061
26–35	1,131	(31.5)	425	(30.8)	706	(32.0)	
36–45	859	(23.9)	289	(20.9)	570	(25.8)	
>45	913	(25.4)	363	(26.3)	550	(24.9)	
Median (IQR)	35	[27–46]	34	[26–46]	36	[27–45]	0.015
Highest level of education							
Primary (or less)	190	(5.3)	30	(2.2)	160	(7.2)	<0.001
Intermediate	423	(11.8)	204	(14.8)	219	(9.9)	
High	672	(18.7)	327	(23.7)	345	(15.6)	
University	1,217	(33.9)	336	(24.3)	881	(39.9)	
Post-graduate	931	(25.9)	401	(29.0)	530	(24.0)	
Marital status							
Single	828	(23.1)	336	(24.3)	492	(22.3)	<0.001
Married	2,466	(68.7)	997	(72.2)	1,469	(66.5)	
Separated/divorced	178	(5.0)	35	(2.5)	143	(6.5)	
Widowed	116	(3.2)	13	(0.9)	103	(4.7)	
Work status							
Employed	2,658	(74.1)	1,063	(77)	1,595	(72.2)	<0.001
Student	255	(7.1)	125	(9.1)	130	(5.9)	
Homemaker	336	(9.4)	0	(0.0)	336	(15.2)	
Retired/unemployed	338	(9.4)	191	(13.8)	147	(6.7)	
Smoking status							
Never	2,784	(77.9)	678	(49.4)	2,106	(95.6)	<0.001
Former	190	(5.3)	163	(11.9)	27	(1.2)	
Current <20 cig/day	164	(4.6)	138	(10.1)	26	(1.2)	
Current 20–39 cig/day	249	(7.0)	243	(17.7)	6	(0.3)	
Current ≥40 cig/day	81	(2.3)	78	(5.7)	3	(0.1)	
Current shisha/pipe/cigar	107	(3.0)	72	(5.2)	35	(1.6)	
Fruits/vegetables (portions/day)							
<1	660	(18.6)	231	(17.0)	429	(19.6)	<0.001
1–1.9	930	(26.2)	316	(23.3)	614	(28.1)	
2–2.9	687	(19.4)	278	(20.5)	409	(18.7)	
3–4.9	703	(19.8)	272	(20.0)	431	(19.7)	
≥5	564	(15.9)	260	(19.2)	304	(13.9)	
Physical activity (hours/week)							
None	2,077	(57.9)	660	(47.8)	1,417	(64.2)	<0.001
0.1–3.0	584	(16.3)	226	(16.4)	358	(16.2)	
3.1–7.0	423	(11.8)	204	(14.8)	219	(9.9)	
>7.0	466	(13.0)	275	(19.9)	191	(8.7)	
Body mass index (kg/m^2^)							
<18.5	41	(1.1)	13	(0.9)	28	(1.3)	<0.001
18.5–24.9	750	(20.9)	271	(19.6)	479	(21.7)	
25–29.9	1,299	(36.2)	580	(42.0)	719	(32.6)	
30.0–34.9	913	(25.4)	344	(24.9)	569	(25.8)	
≥35	586	(16.3)	173	(12.5)	413	(18.7)	
History of cardiovascular disease (% yes)^b^	222	(6.2)	95	(6.9)	127	(5.8)	0.17
History of diabetes (% yes)^c^	400	(11.2)	165	(12.0)	235	(10.6)	0.22

a *Men vs. women*.

b *Self-reported myocardial infarction, angina, or stroke*.

c *Self-reported history of diabetes*.

*P-values for categorical variables were obtained by the Chi-square test, for continuous variables by the Mann-Whitney-U test. Manufactured or hand-rolled cigarettes (cig) were combined*.

**Table 2:** Anthropometric measures by sex and age (weighted^a^ analysis).

			**Age group**							
	**All men (*****n*** **= 1,381)**		**≤25 years (*****n*** **= 304)**		**26–35 years (*****n*** **= 425)**		**36–45 years (*****n*** **= 289)**		**>45 years (*****n*** **= 363)**	
**Men**	**Median**	**[IQR]**	**Median**	**[IQR]**	**Median**	**[IQR]**	**Median**	**[IQR]**	**Median**	**[IQR]**
Height, cm	172	[167–177]	172	[168–177]	174	[169–178]	172	[167–176]	170	[165–175]
Weight, kg	85	[74–95]	80	[70–90]	84	[74–93]	87	[77–98]	86	[78–97]
Body mass index (BMI), kg/m^2^	28.4	[25.5–31.7]	26.7	[23.9–30.1]	27.8	[25.3–30.9]	29.4	[27.1–33.3]	29.8	[27.0–33.4]
Waist circumference, cm	92	[85–102]	86	[77–96]	90	[83–98]	98	[90–105]	100	[90–109]
Hip circumference, cm	105	[98–112]	101	[94–110]	104	[98–111]	107	[100–114]	107	[100–114]
Waist to height ratio	0.54	[0.49–0.60]	0.50	[0.45–0.56]	0.52	[0.48–0.57]	0.57	[0.53–0.62]	0.59	[0.54–0.64]
Waist to hip ratio	0.89	[0.84–0.94]	0.86	[0.80–0.91]	0.87	[0.82–0.92]	0.91	[0.87–0.95]	0.93	[0.89–0.97]
Elevated waist-to-hip ratio, ≥0.90 (n, %)	609	(46.9%)	89	(32.4%)	136	(35.8%)	153	(56.7%)	231	(70.0%)
**Women**	**All women** **[*****n*** **= 2,208]**		**≤25 years** **[*****n*** **= 382]**		**26–35 years** **[*****n*** **= 706]**		**36–45 years** **[*****n*** **= 570]**		**>45 years** **[*****n*** **= 550]**	
Height, cm	158	[154–162]	159	[156–163]	159	[155–163]	159	[155–163]	157	[153–160]
Weight, kg	73	[63–85]	66	[56–76]	70	[62–80]	78	[69–88]	78	[70–88]
Body mass index (BMI), kg/m^2^	29.1	[25.2–33.5]	25.6	[22.7–30.0]	27.4	[24.5–31.2]	30.8	[27.0–35.1]	32.0	[28.5–35.8]
Waist circumference, cm	88	[79–98]	80	[70–90]	83	[76–91]	90	[82–99]	96	[88–103]
Hip circumference, cm	106	[98–115]	102	[93–110]	104	[96–110]	109	[101–117]	110	[102–119]
Waist to height ratio	0.55	[0.49–0.62]	0.50	[0.45–0.56]	0.52	[0.48–0.57]	0.57	[0.52–0.62]	0.61	[0.56–0.67]
Waist to hip ratio	0.79	[0.77–0.89]	0.79	[0.74–0.84]	0.79	[0.75–0.85]	0.83	[0.77–0.88]	0.87	[0.81–0.92]
Elevated waist-to-hip ratio, ≥0.85 (n, %)	783	(37.9%)	79	(22.3)	179	(27.0%)	212	(40.2%)	313	(59.7%)

a *Median values and interquartile range (IQR) are reported (except for elevated waist-to-hip ratios, which are frequencies), weighted by sampling weights to allow population-based estimates. All measures were statistically significantly associated across age group, in men and in women (P_trend_ < 0.001), the p-values for which were obtained from univariate linear regression models between each continuous measure and age group, both analyzed as continuous variables*.

Two multivariable logistic regression models were used to estimate the association of the population characteristics with overweight and obesity (BMI ≥ 25 kg/m^2^) and obesity (BMI ≥ 30 kg/m^2^), included in the models as the binary dependent variables (Table 3). The enter method was used for covariate selection, with each model including the following independent variables: age, health region (estimates not reported), education, marital status, work status, smoking status, fruit, and vegetable consumption, physical activity, self-reported history of myocardial infarction, angina, or stroke and self-reported history of diabetes. Adjusted odds ratios (AORs) and corresponding 95% confidence intervals (CIs) are reported. Linear trends for education, smoking status (excluding those in the category “Current shisha/pipe/cigar”), fruit and vegetable consumption and physical activity were assessed entering those variables as continuous variables in the multivariable logistic regression models. All logistic regression models adhered to the goodness-of-fit test (*p*-value > 0.05). Analysis of variance was used to obtain the weighted age-adjusted means of the anthropometric measures according to participant characteristics (Table 4).

**Table 3:** Multivariable analysis: population characteristics in association with overweight and obesity by sex.

	**Overweight/obesity (BMI ≥ 25)**		**Obesity (BMI ≥ 30)**	
**Characteristic^**a**^**	**Men AOR (95% CI)**	**Women AOR (95% CI)**	**Men AOR (95% CI)**	**Women AOR (95% CI)**
Age (years); +10 year increase	**1.3 (1.1–1.6)**	**1.8 (1.6–2.1)**	**1.3 (1.1–1.5)**	**1.6 (1.4–1.8)**
Education				
Primary	0.4 (0.1–1.1)	0.9 (0.4–2.0)	**0.4 (0.2–0.9)**	1.6 (0.9–2.8)^c^
Intermediate	0.8 (0.5–1.2)	1.5 (0.9–2.5)	0.9 (0.6–1.2)	**1.6 (1.1–2.3)**^c^
High	1.0 (0.7–1.5)	1.2 (0.9–1.8)	1.0 (0.8–1.4)	**1.5 (1.1–2.1)**^c^
University	0.8 (0.5–1.1)	1.2 (0.96–1.6)	0.8 (0.6–1.1)	1.2 (0.9–1.5)^c^
Post-graduate	[Reference]	[Reference]	[Reference]	[Reference]
Marital status				
Single	[Reference]	[Reference]	[Reference]	[Reference]
Married	**1.6 (1.1–2.3)**	**1.7 (1.3–2.2)**	1.0 (0.7–1.5)	1.3 (0.96–1.6)
Separated/divorced	0.8 (0.3–1.8)	1.3 (0.8–2.1)	0.5 (0.2–1.3)	1.2 (0.8–1.9)
Widowed	0.7 (0.2–3.5)	0.8 (0.4–1.6)	0.4 (0.1–1.7)	0.7 (0.4–1.2)
Work status				
Employed	[Reference]	[Reference]	[Reference]	[Reference]
Student	0.7 (0.4–1.2)	0.9 (0.6–1.4)	**0.5 (0.3–0.9)**	0.8 (0.5–1.3)
Homemaker	–	1.3 (0.7–2.3)	–	1.2 (0.8–1.8)
Retired/unemployed	0.8 (0.5–1.3)	1.0 (0.6–1.7)	1.1 (0.8–1.7)	1.3 (0.9–1.9)
Smoking status				
Never	[Reference]	[Reference]	[Reference]	[Reference]
Former	1.1 (0.7–1.7)	0.7 (0.3–1.8)	0.9 (0.6–1.4)^c^	0.9 (0.4–2.1)
Current <20 cig/day	0.9 (0.6–1.4)	0.6 (0.3–1.5)	1.1 (0.7–1.6)^c^	0.5 (0.2–1.2)
Current 20–39 cig/day	0.9 (0.6–1.4)	1.6 (0.2–15.3)	1.3 (0.9–1.8)^c^	1.2 (0.20–6.6)
Current ≥40 cig/day	1.8 (0.8–3.6)	–	**2.0 (1.2–3.3)**^c^	1.7 (0.1–21.0)
Current shisha/pipe/cigar	0.9 (0.5–1.6)	0.9 (0.4–2.1)	1.1 (0.6–1.8)	0.7 (0.3–1.6)
Physical activity (hours/week)				
None	[Reference]	[Reference]	[Reference]	[Reference]
0.1–3.0	0.7 (0.5–1.04)	0.8 (0.6–1.1)	0.8 (0.6–1.1)^c^	0.8 (0.6–1.03)
3.1–7.0	0.9 (0.6–1.4)	1.0 (0.7–1.4)	**0.7 (0.5–0.97)**^c^	1.0 (0.7–1.4)
>7.0	0.7 (0.5–1.1)	0.9 (0.6–1.4)	**0.7 (0.5–0.96)**^c^	1.2 (0.9–1.7)
History of diabetes^b^ (Y vs. N)	**1.8 (1.02–3.3)**	**1.9 (1.1–3.4)**	**1.8 (1.3–2.7)**	**2.1 (1.5–3.0)**

a *Neither obesity nor overweight/obesity were associated with history of heart disease or consumption of fruits and vegetables among men and women. The full tabulation of estimates can be found in Supplemental Table 1*.

b *Self-reported history of diabetes*.

c *Significant linear trend (for smoking the category “Current shisha/pipe/cigar was excluded”)*.

*Values in bold are statistically significant*.

**Table 4:** BMI, waist circumference, waist to height and waist to hip ratio age-adjusted means according to sex and other participant characteristics^a^.

**Variable**	**Men**				**Women**			
	**BMI**	**Waist** **circum**.	**Waist** **to height**	**Waist** **to hip**	**BMI**	**Waist** **circum**.	**Waist** **to height**	**Waist** **to hip**
Education								
Primary	26.5	90.9	0.54	0.93	31.4	94.5	0.60	0.86
Intermediate	29.1	94.5	0.55	0.89	31.3	92.0	0.58	0.85
High	29.4	94.0	0.55	0.89	30.0	88.5	0.56	0.84
University	29.1	93.4	0.54	0.89	29.6	87.5	0.55	0.83
Post-graduate	29.3	95.7	0.56	0.90	28.8	87.1	0.55	0.82
*p*-value	0.12	0.15	0.32	0.06	** <0.01**	** <0.01**	** <0.01**	** <0.01**
Marital status								
Single	28.9	93.5	0.55	0.89	29.3	87.0	0.55	0.82
Married	29.3	95.1	0.55	0.90	30.1	89.2	0.56	0.83
Separated/Divorced	28.1	89.1	0.53	0.89	29.8	89.4	0.57	0.84
Widowed	25.9	79.0	0.46	0.82	28.6	89.5	0.57	0.84
*p*-value	0.07	** <0.01**	** <0.01**	**0.03**	** <0.01**	**0.04**	0.10	0.20
Work status								
Employed	29.3	94.4	0.55	0.89	29.6	87.4	0.55	0.82
Student	27.8	93.0	0.55	0.90	28.6	86.3	0.55	0.82
Homemaker	–	–	-	–	31.5	94.2	0.60	0.85
Retired/unemployed	29.6	95.1	0.56	0.90	29.7	90.3	0.57	0.86
*p*-value	**0.03**	0.57	0.35	0.52	** <0.01**	** <0.01**	** <0.01**	** <0.01**
History of diabetes^b^								
No	29.0	93.9	0.56	0.89	29.5	87.7	0.55	0.83
Yes	30.5	98.1	0.58	0.91	32.1	96.2	0.61	0.87
*p*-value	**0.01**	** <0.01**	** <0.01**	**0.01**	** <0.01**	** <0.01**	** <0.01**	** <0.01**

*Age-adjusted means are reported, weighted by sampling weights to allow population-based estimates*.

a *The anthropometric measures were not consistently associated with smoking status, consumption of fruits/vegetables, physical activity, or history of heart disease among men and women. The full tabulation of estimates can be found in Supplemental Table 2*.

b *Self-reported history of diabetes*.

*Values in bold are statistically significant*.

### Ethical Considerations

The study followed the principles of the Declaration of Helsinki and was approved by the MOH Standing Committee for the Coordination of Medical and Health Research. Written informed consent was obtained from each participant prior to enrolment in the study after explanation of the study procedures.

## Results

The median age of participants was 35 years and the proportion of women was 61.5% (Table 1). The weighted mean age was 36.3 (±12.3) years. There was a similar distribution of age among men and women. Compared to men, a larger proportion of women were university graduates (39.9 vs. 24.3%), homemakers (15.2 vs. 0%), never smokers (95.6 vs. 49.4%), sedentary (64.2 vs. 47.8%), and with BMI ≥ 35 kg/m^2^ (18.7 vs. 12.5%). More men than women were married (72.2 vs. 66.5%) and ate five or more portions of fruits and vegetables per day (19.2 vs. 13.9%). There was a similar proportion of men and women with self-reported previous history of heart diseases (total 6.2%) and diabetes (total 11.2%).

Detailed anthropometric measures including medians and inter quartile ranges by sex and age are presented in Table 2. All measures were statistically significantly associated with age in men and women (*p* for trend <0.001, with *p*-values for trend obtained from univariate linear regression models between each measure and age category, both analyzed as continuous variables). Among men, the median WHtR was 0.54, median WHiR was 0.89, and median BMI was 28.4 kg/m^2^. Among women, the median WHtR was 0.55, the WHiR was 0.79 and median BMI was 29.1 kg/m^2^. Overall, the mean BMI was 29.4 ± 6.0 kg/m^2^ (29.8 ± 7.0 among women and 29.1 ± 5.0 among men; *p* < 0.001).

**Figure 1:**
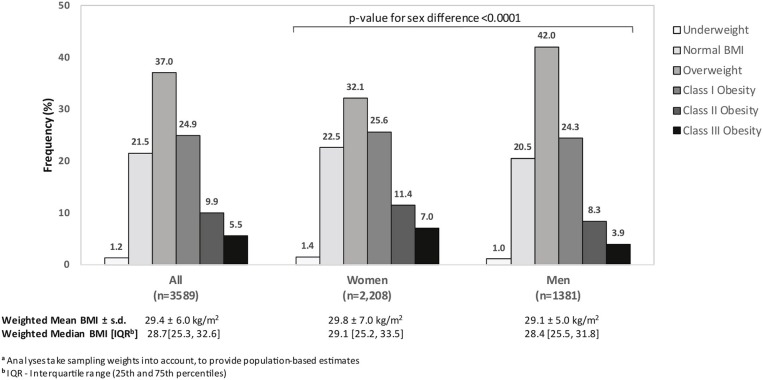
Weighted^a^ distribution of WHO body mass index (BMI) category among Kuwaiti citizens, according to sex, STEPS Study, 2014.

The weighted prevalence of obesity was 40.3% [95% confidence interval, 38.6–42.0%] (Figure 1). The weighted prevalence of Class I obesity was 24.9% [23.5–26.4%], Class II obesity was 9.9% [8.9–10.9%], and Class III was 5.5% [4.8–6.3%] in Kuwaiti adults. The prevalence of overweight was 37.0% [35.4–38.7%]. Amongst Kuwaiti men, the prevalence of obesity was 36.5% (24.3, 8.3, and 3.9% in Class I, II, and III, respectively) and the prevalence of obesity amongst Kuwaiti women was 44.0% (25.6, 11.4, and 7.0% in Class I, II, and III, respectively). The weighted prevalence of overweight was 42.0% in men and 32.1% in women. Only 22.7% of Kuwaiti adults had a BMI of <25 kg/m^2^ (23.9% of women and 21.5% of men). The combined prevalence of overweight and obesity was 77.3% [75.8, 78.7] (Men: 78.5%; Women: 76.1%).

**Figure 2:**
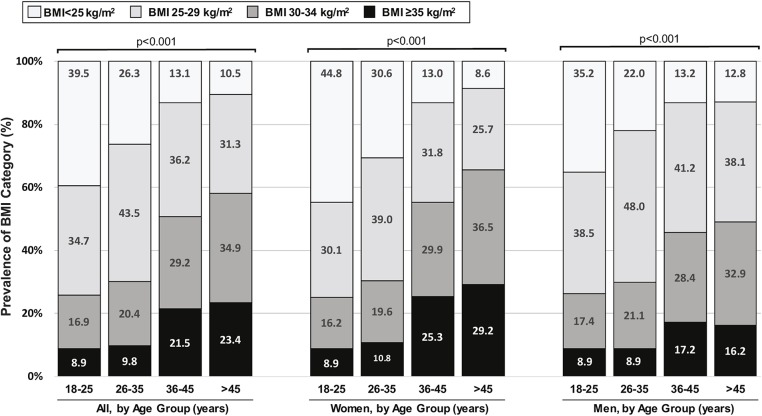
Weighted^a^ distribution of WHO body mass index (BMI) category among Kuwaiti citizens, according to age group and sex, STEPS Study, 2014. ^*a*^Analyses take sampling weights into account, to provide population-based estimates.

Age was strongly associated with BMI (Figure 2). The prevalence of obesity was 25.8% in the 18–25-year group, compared with 58.3% in people aged 45 years or over and the prevalence of overweight and obesity combined from 60.5 to 89.6%, in these age groups, respectively. This strong statistically significant (*p* < 0.001) trend across age was seen in both men and women.

Multivariable analysis of population characteristics associated with overweight and obesity by sex are presented in Table 3 and Supplementary Table 1. Age was significantly associated with an increased prevalence of overweight and obesity in both sexes. Men with primary education only were less obese than men with post-graduate education, while among women there was a significant linear inverse trend of obesity with educational levels. Married men and women had significant increased odds of being overweight or obese when compared with single individuals. Current smoking of ≥40 cigarettes per day was associated with obesity in men compared with those never smoking. A significant inverse linear trend was found between physical activity (hours/week) and obesity in men but not in women. The self-reported history of diabetes was significantly associated with overweight and obesity in both sexes.

Table 4 and Supplementary Table 2 show age-adjusted means, weighted by sampling weights to allow population-based estimates, for selected anthropometric measures, according to other participants characteristics. Among women, but not among men, there was a statistically significant inverse linear association of all these anthropometric measures with education. Analysis by occupational status did not reveal any patterns among men, while it confirmed that women who were homemakers had higher mean BMI, waist circumference, WHiR, and WHtR than other occupational categories. There were no clear associations between adiposity indicators and smoking among men, but women currently smoking ≥40 cigarettes per day had a higher WHiR (1.00) than other women. Physically active men had a significantly lower WHiR and WHtR while such association was not seen in women. Self-reported history of diabetes was significantly associated with higher anthropometric measurements (all variables) for men and women, while self-reported history of heart diseases was associated with higher waist circumference and WHtR in women only.

## Discussion

This cross-sectional survey found that among Kuwaiti adults the prevalence of overweight and obesity are extremely high and rank among the highest reported throughout the world. Only 22% of men and 24% of women had a BMI <25 kg/m^2^, while 37% of men and 44% of women were obese. In both sexes, the prevalence of overweight and obesity were associated with increasing age, being married and self-reported diabetes. Amongst men, having a low educational level, being employed, currently smoking ≥40 cigarettes per day and being physically inactive were associated with either overweight and obesity. Among women there was a lower prevalence of obesity with increasing education level, while there were no clear associations with other additional characteristics studied.

The importance of such a high prevalence of overweight and obesity on public health in Kuwait, both now and in the future, cannot be overstated. The high obesity prevalence has undoubtedly contributed substantially to the 19% prevalence of diabetes reported among Kuwait adults, and the 63% prevalence in those aged 60–69 years (6). However, diabetes is only one of the subsequent waves of longer-term comorbidities to result from increased obesity prevalence. Over time, concomitant increases will likely follow in the incidence of hypertension (8), cardiovascular disease (9), cognitive decline (10), and many cancers (11). The prevalence of risk factors is directly proportional to the population attributable risk (PAR) of outcomes. With a prevalence of overweight and obesity above 75% in the adult Kuwaiti population, it will contribute to an increasing percentage of PAR over time, even for outcomes for which obesity has weak to modest associations.

Also of great public health importance is our finding of ~60% prevalence of overweight and obesity in participants aged 18–25, suggesting that the majority of overweight and obesity in Kuwait has already become established by the time adulthood is reached. One of the main policy implications of this finding is that the focus of public health efforts must be directed to children and adolescents. This is consistent with the recommendations of the World Health Organization that “developing coherent public policies from production to consumption and across relevant sectors, through forming a cross-governmental task force, to oversee the development and/or strengthening of policies to ensure healthy diets throughout the life-course” (12). Behavioral interventions, together with public policies, regulation of industry and health education, will be necessary to stem the tide of increasing obesity (13). The Kuwait MOH and the Kuwait Public Authority for Food and Nutrition are both actively focusing on the issue of childhood obesity within their own domains. Such programs need to be supported, expanded to other stakeholders and linked together, as it is evident that a substantial reduction in adult obesity can only occur by addressing the obesity epidemic at earlier ages.

A few other population-based surveys reporting obesity estimates in the Kuwaiti population have been conducted in Kuwait since 2001. The estimates of overweight, obesity and combined overweight and obesity, respectively, were reported as follows: in 2001, estimates of 35, 24, and 58% (from a household survey of 7,609 Kuwaiti nationals aged 15–84 years) (14); in 2006, estimates of 34, 42, and 75% (from the first STEPS survey conducted among 2,280 Kuwaitis aged 20–64 years) (4); in 2008–9, estimates of 33, 43, and 76% (a national nutrition household survey of 1,704 Kuwaitis aged 19–86 years) (15); and in 2013, estimates of 38, 37, and 75% (a World Health Survey household survey of 2,995 Kuwaiti households) (16). The prevalence estimates of overweight, obesity, and overweight and obesity combined in the present study were 37, 40, and 77%.

The 2001 household survey estimates should not be directly compared to the other studies referenced above largely because one-third of the 2001 sample was between age 15 and 24 years, thus lowering the prevalence estimates compared with our study by an unknown amount, and the study did not use population sampling weights. Two other studies which analyzed the 2006 STEPS study data (17, 18) reported higher prevalence estimates than the official 2006 Kuwait STEPS monograph itself. However, both studies may have overestimated the true population prevalence since the mean age in the sample was substantially higher than that of the target population, and the study analyses did not utilize the population sampling weights. When unweighted estimates are used, they reflect the prevalence in the sample itself and do not necessarily provide accurate inferences for the target population. A comparison of the estimates from the four surveys between 2006 and 2014 indicates a slight increase in prevalence of overweight and obesity combined during this 8-year period from 75 to 77%. However, caution is warranted when making direct comparisons and inferences regarding temporal trends.

The estimates of the prevalence of overweight and obesity are high across the Arabian Gulf countries (3). Several risk factors likely explain the high prevalence of overweight and obesity in the region. These include genetic susceptibility, diet with excessive energy, low physical activity and cultural barriers for physical activity (19). Recent decades were marked by rapid urbanization and economic development as well as changes in traditional cultural factors in Kuwait, resulting in the adoption of more sedentary lifestyles and high energy intake diets (15), contributing to the increasing prevalence of overweight and obesity.

As expected, consistent age-adjusted direct associations of the anthropometric measures with diabetes were seen in both men and women. Consistent associations of the measures with being married were present primarily in men, and less consistently among women suggesting that marriage, especially for men, may be associated with dietary changes. Future qualitative studies could explore this finding further and provide evidence for health education programs that target families. We also found consistent inverse associations with education and direct associations with being a homemaker among women (no men identified as homemakers). These findings also can be used to inform health education programs that target women's dietary knowledge and practices. No consistent associations of the anthropometric measures were seen with smoking, fruit and vegetable consumption, physical activity, or cardiovascular disease (Supplementary Table 1). There were a few scattered statistically significant associations of specific fatness measures with these characteristics, but no firm inferences should be made due to the possibility of chance findings due to the multiple comparisons.

There is no consensus about which anthropometric parameter best predicts diabetes or other NCDs (20–22). The association of diabetes with increasing BMI has been well-established (23, 24), as is the validity of BMI in predicting body fat mass and morbidity (25). However, BMI is an imperfect measurement of adiposity as it does not distinguish between lean and fat tissue (25), and has a J-shaped association with cardiovascular mortality and all-cause mortality, a phenomena known as the “obesity paradox” (26). In contrast, waist circumference, WHiR, and WHtR all predict diabetes incidence, cardiovascular mortality and all-cause mortality more directly (9, 27, 28). Nevertheless, the significant and consistent associations of the various anthropometric characteristics and self-reported diabetes suggest that measuring body fatness—regardless of the technique used—may be useful in identifying people at risk of diabetes.

An inverse association between smoking and obesity among men has been reported in other populations (29). In a previous study in Kuwait, smoking was associated with decreased risk of overweight but not obesity (30). In contrast, we found smoking ≥40 cigarettes a day increased obesity prevalence in men. Among women, an association between smoking and anthropometric measures was captured but only when using WHiR, which was highest amongst smokers of 40 or more cigarettes per day. This is consistent with findings from other studies that have reported higher waist circumference and WHiR in smokers than in non-smokers (31, 32). In contrast, a study on smoking and obesity among women in the USA reported smokers had decreased odds of being overweight or obese (33).

The strengths of this study include the population-based sampling design, use of the internationally-accepted methods of the WHO STEPS studies and a sufficiently large sample size for the purpose of a cross-sectional NCD risk factor survey. The study also had some limitations. This survey included only Kuwaiti nationals, who make up <30% of the country's total population of nearly 4.5 million (34). Future population health surveys in Kuwait should sample both national and non-national residents in Kuwait to provide data for the entire population living in the country. A previous study reported that prevalence of overweight and obesity amongst non-Kuwaitis living in Kuwait was lower than that amongst Kuwaiti nationals (35). Another limitation is that the study did not reach the target sample size, which was calculated to achieve a 5% margin of error in obesity prevalence within each of the eight sex-age categories. Therefore, the margin of error around estimates may be wider than 5%, and comparisons of binary data among these groups may fail to detect some weaker associations that are present. However, the power for detecting associations with continuous variables (e.g., WHiR, etc.) is substantially higher, since the power for parametric statistics is greater for continuous variables than it is for binary variables.

In conclusion, our findings highlight the major challenge facing Kuwait, similarly to other countries in the Gulf region, in controlling the obesity epidemic in this region. Being overweight or obese is the norm rather than an exception amongst Kuwaitis. Public health action to decrease overweight and obesity in Kuwait are urgently needed. The long-term health consequences and economic burden of obesity in the Kuwaiti society and health care system will likely be overwhelming in the decades to come. Recommendations on physical activity and healthy diet were discussed at the conference on Healthy Lifestyles and Non-Communicable Diseases in the Arab World and the Middle East in 2012 and included in the Riyadh declaration (36), but these recommendations have not yet been fully implemented.

As Kuwaiti adults rank amongst the highest for diabetes burden in the world, life-course weight waist circumference monitoring may be considered as a population-level intervention. Primary health care should aim at helping individuals avoid becoming obese, or losing weight, as needed. Monitoring of body weight and waist circumference should necessarily be followed by nutritional and lifestyle education and support.

## Ethics Statement

This study was carried out in accordance with the recommendations of Ministry of Health Standing Committee for Health Research, with written informed consent from all subjects. All subjects gave written informed consent in accordance with the Declaration of Helsinki. The protocol was approved by the Ministry of Health Standing Committee for Health Research.

## Author Contributions

RA-W and QA organized and coordinated the data collection. EW, EB, and JL performed the data analysis. EW, EB, JL, AA, and JT contributed to the interpretation of the data and drafting the article. All authors were involved in the critical evaluation and final approval of the article.

### Conflict of Interest Statement

All authors contributed to the conceptual design of this study. Where authors are identified as personnel of the International Agency for Research on Cancer/World Health Organization, the authors alone are responsible for the views expressed in this article and they do not necessarily represent the decisions, policy or views of the International Agency for Research on Cancer/World Health Organization.
