# Supplementary material for: The Prevalence of Overweight and Obesity in an Adult Kuwaiti Population in 2014
Source: Front Endocrinol (Lausanne). 2019 Jul 9;10:449. doi: 10.3389/fendo.2019.00449 (PMC6629831; doi:10.3389/fendo.2019.00449)
Supplement: Supplementary file 1 [file Data_Sheet_1.doc]

**Supplemental Table 1:** Multivariable analysis: population characteristics in association with overweight and obesity by sex (complete)

|  | **Overweight/Obesity**  (BMI ≥ 25) | |  | **Obesity**  (BMI ≥ 30) | |
| --- | --- | --- | --- | --- | --- |
| **Characteristic** | **Men**  **AOR (95% CI)** | **Women**  **AOR (95% CI)** |  | **Men**  **AOR (95% CI)** | **Women**  **AOR (95% CI)** |
| Age (years); +10 year increase | **1.3 (1.1-1.6)** | **1.8 (1.6-2.1)** |  | **1.3 (1.1-1.5)** | **1.6 (1.4-1.8)** |
|  |  |  |  |  |  |
| Education |  |  |  |  |  |
| Primary | 0.4 (0.1-1.1) | 0.9 (0.4-2.0) |  | **0.4 (0.2-0.9)** | 1.6 (0.9-2.8) ^c^ |
| Intermediate | 0.8 (0.5-1.2) | 1.5 (0.9-2.5) |  | 0.9 (0.6-1.2) | **1.6 (1.1-2.3)** ^c^ |
| High | 1.0 (0.7-1.5) | 1.2 (0.9-1.8) |  | 1.0 (0.8-1.4) | **1.5 (1.1-2.1)** ^c^ |
| University | 0.8 (0.5-1.1) | 1.2 (0.96-1.6) |  | 0.8 (0.6-1.1) | 1.2 (0.9-1.5) ^c^ |
| Post-graduate | [Reference] | [Reference] |  | [Reference] | [Reference] |
|  |  |  |  |  |  |
| Marital status |  |  |  |  |  |
| Single | [Reference] | [Reference] |  | [Reference] | [Reference] |
| Married | **1.6 (1.1-2.3)** | **1.7 (1.3-2.2)** |  | 1.0 (0.7-1.5) | 1.3 (0.96-1.6) |
| Separated/Divorced | 0.8 (0.3-1.8) | 1.3 (0.8-2.1) |  | 0.5 (0.2-1.3) | 1.2 (0.8-1.9) |
| Widowed | 0.7 (0.2-3.5) | 0.8 (0.4-1.6) |  | 0.4 (0.1-1.7) | 0.7 (0.4-1.2) |
|  |  |  |  |  |  |
| Work status |  |  |  |  |  |
| Employed | [Reference] | [Reference] |  | [Reference] | [Reference] |
| Student | 0.7 (0.4-1.2) | 0.9 (0.6-1.4) |  | **0.5 (0.3-0.9)** | 0.8 (0.5-1.3) |
| Homemaker | - | 1.3 (0.7-2.3) |  | - | 1.2 (0.8-1.8) |
| Retired/Unemployed | 0.8 (0.5-1.3) | 1.0 (0.6-1.7) |  | 1.1 (0.8-1.7) | 1.3 (0.9-1.9) |
|  |  |  |  |  |  |
| Smoking status |  |  |  |  |  |
| Never | [Reference] | [Reference] |  | [Reference] | [Reference] |
| Former | 1.1 (0.7-1.7) | 0.7 (0.3-1.8) |  | 0.9 (0.6-1.4) ^c^ | 0.9 (0.4-2.1) |
| Current < 20 cig / day | 0.9 (0.6-1.4) | 0.6 (0.3-1.5) |  | 1.1 (0.7-1.6) ^c^ | 0.5 (0.2-1.2) |
| Current 20-39 cig / day | 0.9 (0.6-1.4) | 1.6 (0.2-15.3) |  | 1.3 (0.9-1.8) ^c^ | 1.2 (0.20-6.6) |
| Current ≥ 40 cig / day | 1.8 (0.8-3.6) | - |  | **2.0 (1.2-3.3)** ^c^ | 1.7 (0.1-21.0) |
| Current shisha/pipe/cigar | 0.9 (0.5-1.6) | 0.9 (0.4-2.1) |  | 1.1 (0.6-1.8) | 0.7 (0.3-1.6) |
|  |  |  |  |  |  |
| Fruit/vegetables (Courses / day) |  |  |  |  |  |
| <1 | [Reference] | [Reference] |  | [Reference] | [Reference] |
| 1-1.9 | 1.3 (0.8-2.0) | 1.34 (0.99-1.8) |  | 1.3 (0.9-1.9) | 1.0 (0.8-1.3) |
| 2-2.9 | 1.1 (0.7-1.7) | 1.20 (0.9-1.7) |  | 1.1 (0.7-1.6) | 1.0 (0.7-1.3) |
| 3-4.9 | 1.0 (0.6-1.6) | 1.24 (0.9-1.8) |  | 1.1 (0.8-1.7) | 1.1 (0.8-1.4) |
| ≥ 5 | 1.3 (0.8-2.1) | 0.97 (0.77-1.4) |  | 0.9 (0.6-1.4) | 1.0 (0.7-1.3) |
|  |  |  |  |  |  |
| Physical activity (Hours / week) |  |  |  |  |  |
| None | [Reference] | [Reference] |  | [Reference] | [Reference] |
| 0.1-3.0 | 0.7 (0.5-1.04) | 0.8 (0.6-1.1) |  | 0.8 (0.6-1.1) ^c^ | 0.8 (0.6-1.03) |
| 3.1-7.0 | 0.9 (0.6-1.4) | 1.0 (0.7-1.4) |  | **0.7 (0.5-0.97)** ^c^ | 1.0 (0.7-1.4) |
| > 7.0 | 0.7 (0.5-1.1) | 0.9 (0.6-1.4) |  | **0.7 (0.5-0.96)** ^c^ | 1.2 (0.9-1.7) |
|  |  |  |  |  |  |
| History of heart disease^a^ (Y vs. N) | 0.8 (0.4-1.44) | 1.5 (0.8-2.8) |  | 0.9 (0.6-1.5) | 1.3 (0.9-2.0) |
|  |  |  |  |  |  |
| History of diabetes^b^ (Y vs. N) | **1.8 (1.02-3.3)** | **1.9 (1.1-3.4)** |  | **1.8 (1.3-2.7)** | **2.1 (1.5-3.0)** |

^a^ Self-reported myocardial infarction, angina, or stroke. ^b^ Self-reported history of diabetes. ^c^ Significant linear trend (for smoking the category “Current shisha/pipe/cigar was excluded”)

**Supplemental Table 2:** BMI, waist circumference, waist to height and waist to hip ratio age-adjusted means according to sex and other participant characteristics (complete)

|  | **Men** | | | |  | **Women** | | | |
| --- | --- | --- | --- | --- | --- | --- | --- | --- | --- |
| **Variable** | **BMI** | **Waist**  **circum.** | **Waist to**  **height** | **Waist to**  **hip** |  | **BMI** | **Waist**  **circum.** | **Waist to**  **height** | **Waist to**  **hip** |
| Education |  |  |  |  |  |  |  |  |  |
| Primary | 26.5 | 90.9 | 0.54 | 0.93 |  | 31.4 | 94.5 | 0.60 | 0.86 |
| Intermediate | 29.1 | 94.5 | 0.55 | 0.89 |  | 31.3 | 92.0 | 0.58 | 0.85 |
| High | 29.4 | 94.0 | 0.55 | 0.89 |  | 30.0 | 88.5 | 0.56 | 0.84 |
| University | 29.1 | 93.4 | 0.54 | 0.89 |  | 29.6 | 87.5 | 0.55 | 0.83 |
| Post graduate | 29.3 | 95.7 | 0.56 | 0.90 |  | 28.8 | 87.1 | 0.55 | 0.82 |
| p-value | 0.12 | 0.15 | 0.32 | 0.06 |  | **<0.01** | **<0.01** | **<0.01** | **<0.01** |
|  |  |  |  |  |  |  |  |  |  |
| Marital status |  |  |  |  |  |  |  |  |  |
| Single | 28.9 | 93.5 | 0.55 | 0.89 |  | 29.3 | 87.0 | 0.55 | 0.82 |
| Married | 29.3 | 95.1 | 0.55 | 0.90 |  | 30.1 | 89.2 | 0.56 | 0.83 |
| Separated/Divorced | 28.1 | 89.1 | 0.53 | 0.89 |  | 29.8 | 89.4 | 0.57 | 0.84 |
| Widowed | 25.9 | 79.0 | 0.46 | 0.82 |  | 28.6 | 89.5 | 0.57 | 0.84 |
| p-value | 0.07 | **<0.01** | **<0.01** | **0.03** |  | **<0.01** | **0.04** | 0.10 | 0.20 |
|  |  |  |  |  |  |  |  |  |  |
| Work status |  |  |  |  |  |  |  |  |  |
| Employed | 29.3 | 94.4 | 0.55 | 0.89 |  | 29.6 | 87.4 | 0.55 | 0.82 |
| Student | 27.8 | 93.0 | 0.55 | 0.90 |  | 28.6 | 86.3 | 0.55 | 0.82 |
| Homemaker | - | - | - | - |  | 31.5 | 94.2 | 0.60 | 0.85 |
| Retired/Unemployed | 29.6 | 95.1 | 0.56 | 0.90 |  | 29.7 | 90.3 | 0.57 | 0.86 |
| p-value | **0.03** | 0.57 | 0.35 | 0.52 |  | **<0.01** | **<0.01** | **<0.01** | **<0.01** |
|  |  |  |  |  |  |  |  |  |  |
| Smoking status |  |  |  |  |  |  |  |  |  |
| Never | 29.2 | 94.2 | 0.55 | 0.90 |  | 29.8 | 88.8 | 0.56 | 0.83 |
| Former | 29.1 | 94.3 | 0.55 | 0.89 |  | 29.6 | 87.3 | 0.56 | 0.79 |
| Current < 20 cig/day | 28.4 | 93.4 | 0.54 | 0.90 |  | 28.2 | 84.3 | 0.53 | 0.79 |
| Current 20-39 cig/day | 29.2 | 94.3 | 0.54 | 0.89 |  | 31.6 | 93.4 | 0.56 | 0.84 |
| Current ≥ 40 cig/day | 30.6 | 97.5 | 0.56 | 0.90 |  | 32.8 | 88.3 | 0.54 | 1.00 |
| Cur shisha/pipe/cigar | 28.7 | 95.4 | 0.56 | 0.91 |  | 30.1 | 87.5 | 0.55 | 0.81 |
| p-value | 0.17 | 0.44 | 0.45 | 0.42 |  | 0.67 | 0.54 | 0.54 | **0.02** |
|  |  |  |  |  |  |  |  |  |  |
| Fruit/vegetables (Courses/day) |  |  |  |  |  |  |  |  |  |
| < 1 | 28.9 | 93.9 | 0.55 | 0.89 |  | 30.0 | 89.3 | 0.57 | 0.83 |
| 1-1.9 | 29.3 | 95.4 | 0.56 | 0.90 |  | 29.8 | 88.4 | 0.56 | 0.83 |
| 2-2.9 | 29.1 | 94.2 | 0.55 | 0.90 |  | 30.0 | 89.4 | 0.56 | 0.83 |
| 3-4.9 | 29.1 | 93.4 | 0.54 | 0.90 |  | 30.0 | 88.6 | 0.56 | 0.84 |
| ≥ 5 | 29.2 | 94.7 | 0.55 | 0.89 |  | 29.2 | 87.9 | 0.55 | 0.83 |
| p-value | 0.94 | 0.53 | 0.43 | 0.34 |  | 0.37 | 0.53 | 0.18 | 0.58 |
|  |  |  |  |  |  |  |  |  |  |
| Physical activity |  |  |  |  |  |  |  |  |  |
| None | 29.4 | 94.5 | 0.55 | 0.90 |  | 30.0 | 89.0 | 0.56 | 0.83 |
| 0.1-3.0 | 29.2 | 95.6 | 0.56 | 0.90 |  | 29.4 | 87.6 | 0.55 | 0.83 |
| 3.1-7.0 | 28.6 | 94.5 | 0.55 | 0.89 |  | 29.4 | 87.8 | 0.55 | 0.83 |
| > 7.0 | 28.9 | 92.8 | 0.54 | 0.88 |  | 30.1 | 89.6 | 0.57 | 0.83 |
| p-value | 0.33 | 0.16 | **0.05** | **0.02** |  | 0.28 | 0.21 | 0.30 | 0.99 |
|  |  |  |  |  |  |  |  |  |  |
| History of cardiovascular disease^a^ |  |  |  |  |  |  |  |  |  |
| No | 29.2 | 94.5 | 0.55 | 0.90 |  | 29.8 | 88.5 | 0.56 | 0.83 |
| Yes | 28.4 | 92.9 | 0.54 | 0.89 |  | 30.8 | 91.3 | 0.58 | 0.84 |
| p-value | 0.18 | 0.34 | 0.25 | 0.50 |  | 0.06 | **0.02** | **0.02** | 0.45 |
|  |  |  |  |  |  |  |  |  |  |
| History of diabetes^b^ |  |  |  |  |  |  |  |  |  |
| No | 29.0 | 93.9 | 0.56 | 0.89 |  | 29.5 | 87.7 | 0.55 | 0.83 |
| Yes | 30.5 | 98.1 | 0.58 | 0.91 |  | 32.1 | 96.2 | 0.61 | 0.87 |
| p-value | **0.01** | **<0.01** | **<0.01** | **0.01** |  | **<0.01** | **<0.01** | **<0.01** | **<0.01** |
|  |  |  |  |  |  |  |  |  |  |

Age-adjusted means are reported, weighted by sampling weights to allow population-based estimates ^a^ Self-reported myocardial infarction, angina, or stroke. ^b^ Self-reported history of diabetes.
